# AlnC: An extensive database of long non-coding RNAs in angiosperms

**DOI:** 10.1371/journal.pone.0247215

**Published:** 2021-04-14

**Authors:** Ajeet Singh, A. T. Vivek, Shailesh Kumar

**Affiliations:** Bioinformatics Lab, National Institute of Plant Genome Research (NIPGR), Aruna Asaf Ali Marg, New Delhi, India; ICAR - National Research Center on Plant Biotechnology, INDIA

## Abstract

Long non-coding RNAs (lncRNAs) are defined as transcripts of greater than 200 nucleotides that play a crucial role in various cellular processes such as the development, differentiation and gene regulation across all eukaryotes, including plant cells. Since the last decade, there has been a significant rise in our understanding of lncRNA molecular functions in plants, resulting in an exponential increase in lncRNA transcripts, while these went unannounced from the major Angiosperm plant species despite the availability of large-scale high throughput sequencing data in public repositories. We, therefore, developed a user-friendly, open-access web interface, AlnC (**A**ngiosperm **ln**cRNA **C**atalogue) for the exploration of lncRNAs in diverse Angiosperm plant species using recent 1000 plant (**1KP**) trancriptomes data. The current version of AlnC offers 10,855,598 annotated lncRNA transcripts across 682 Angiosperm plant species encompassing 809 tissues. To improve the user interface, we added features for browsing, searching, and downloading lncRNA data, interactive graphs, and an online BLAST service. Additionally, each lncRNA record is annotated with possible small open reading frames (sORFs) to facilitate the study of peptides encoded within lncRNAs. With this user-friendly interface, we anticipate that AlnC will provide a rich source of lncRNAs for small-and large-scale studies in a variety of flowering plants, as well as aid in the improvement of key characteristics in relevance to their economic importance. Database URL: http://www.nipgr.ac.in/AlnC

## 1. Introduction

Angiosperms are flowering plants that constitute an exceptionally large group of plants that grow in a wide variety of habitats. It comprises of more than 3,000,000 recorded species worldwide, encompassing one of the most diverse group within the plant kingdom [1, 2]. Most angiosperms are a main source of consumer goods like textile fibres, herbs and spices, fuel and pharmaceuticals, as well as a major source of food. Most model plants belonging to this group have been intensively studied to understand flowering and other major mechanisms. Consequently, research in Angiosperms exploded with the advent of next generation sequencing (NGS) resulting in an improved picture of transcriptome, especially from the point of non-coding RNAs (ncRNAs). In recent years, lncRNAs are a major RNA class of greater research interest to study alongside miRNAs. LncRNAs are typically more than 200 nucleotides with no or less protein coding capacity, as several recent studies suggest the presence of sORFs with a potential for translating into micropeptides [3–5]. Further, there is compelling evidence that lncRNAs role in multiple plant biochemical pathways in recent years [6–8]. With the rise of transcriptome data in public repositories, thousands of plant lncRNAs were identified and maintained in a number of lncRNA dedicated databases in the last decade [9–14]. However, despite being in the spotlight, lncRNAs still need to be annotated in a variety of plant species, given the availability of lncRNA databases focused primarily on model plants and major crops. As information on lncRNA in many Angiosperms is still scarce, the advancement of lncRNA research in these plants is largely hindered. This research gap can be potentially addressed by the use of large volumes of RNA sequencing (RNA-seq) data available in public databases as it provides enormous opportunities to discover and classify potential lncRNAs [15, 16]. Various lncRNA investigations in plant species were undertaken using independent bioinformatics pipelines for genome-wide annotation and further, archiving in databases, but several plants are still unexplored due to a complete lack of complete genome sequences [15, 17, 18]. In order to fill this potential gap, various lncRNA methods are available and are increasingly being developed to improve lncRNA identification and annotation from *de novo* assembled transcripts [19, 20].

In this post-genomics era/advanced genomics era, it is crucial to develop comprehensive methods for lncRNA annotation across various plant species to improve our understanding of the complex interplay of lncRNAs across plants. More importantly, the creation and maintenance of a stable lncRNA data repository is equally necessary if lncRNA biology is to be understood [17, 21]. In this research work, we use large-scale transcriptome data to identify potential lncRNAs with three major goals. First, we anticipate to provide information on most potential lncRNAs of plant species with no available genome sequence. The second goal is to mediate the importance of unused large-scale datasets as an annotation source for thousands of lncRNAs. Last, to develop a database that can act as a catalyst to promote lncRNA research in angiosperms and to provide a one-stop data access platform. Here, we capitalised on large-scale transcriptomic data of 682 angiosperms from the 1000 plants (1KP) project as this enabled us to annotate 1,08,55,598 lncRNAs. The results of the study were organised and stored in a user-friendly web-interface, the AlnC) with a plan to update periodically on the basis of new knowledge and an expansion in the number of species of Angiosperm in the future.

## 2. Materials and methods

### Data collection and systematic lncRNA identification

**Fig 1:**
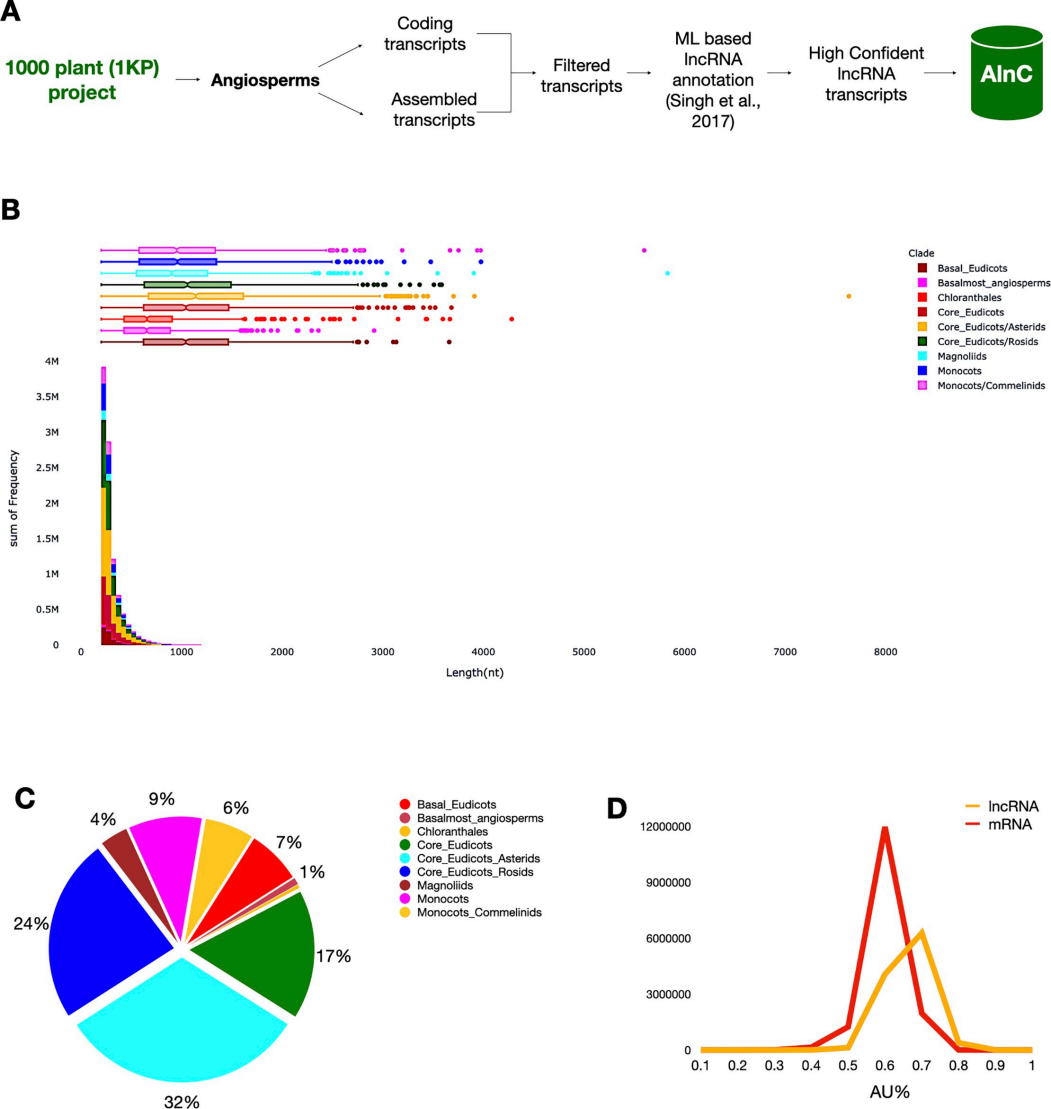
Overview of lncRNAs in AlnC. (A) Systematic workflow adopted to annotate potential lncRNAs of flowering plants available from the 1KP project. (B) Length-wise distribution of AlnC annotated lncRNAs across clades. (C) Pie chart represents the percentage of lncRNA entries in AlnC. (D) Percentage composition of AU content in protein-coding transcripts and lncRNAs in AlnC.

To find potential lncRNAs, we used *de novo* assembled transcripts of 682 Angiosperm plant species from the 1KP project (http://www.onekp.com/public_data.html) [22]. For each species, lncRNAs were identified from each sample relying on a bioinformatics pipeline previously exploited by Singh et al., 2017, and transcripts longer than 200 bp were retained that are not overlapping with protein-coding gene models (Fig 1(A)) [23]. First, we excluded potential coding transcripts from the assembled transcripts set for that species (translated proteins of matched orthogroups derived from annotated plant genomes) [24]. Further, protein-coding transcripts were discarded using PLncPRO (python prediction.py -p plncpro -result-file -i sequence.fa -m models/<monocot or dicot>.model -o plncpro-out -d lib/blastdb/swiss-protDB -t 15 -r) on the basis of a BLAST approach to a manually curated list of Swissprot proteins [25]. In the final filtering step, high-confident lncRNA transcripts were extracted by setting a minimum length threshold of 200 nt length and a non-coding probability score of 0.8 (python predstoseq.py -f sequence.fa -o output-file -p plncpro-result-file -l 0 -s 0.8—min 200).

### Database construction and implementation

AlnC is operating on a Linux, Apache, MySQL and PHP stack as of now. The current database framework is built on the Apache server and the AlnC web interface has been designed using HTML, CSS, and JavaScript. All AlnC lncRNAs and other related data annotations is handled by a relational database set up with MySQL. In addition, AlnC is integrated with stand-alone BLAST (v2.11.0) for online similarity search, ViennaRNA (v2.4.16) for the visualisation of secondary structure and ORFfinder (v0.4.3) for the exploration of lncRNA containing sORFs [26–28].

## 3. Results and discussion

### Data content in AlnC

**Fig 2:**
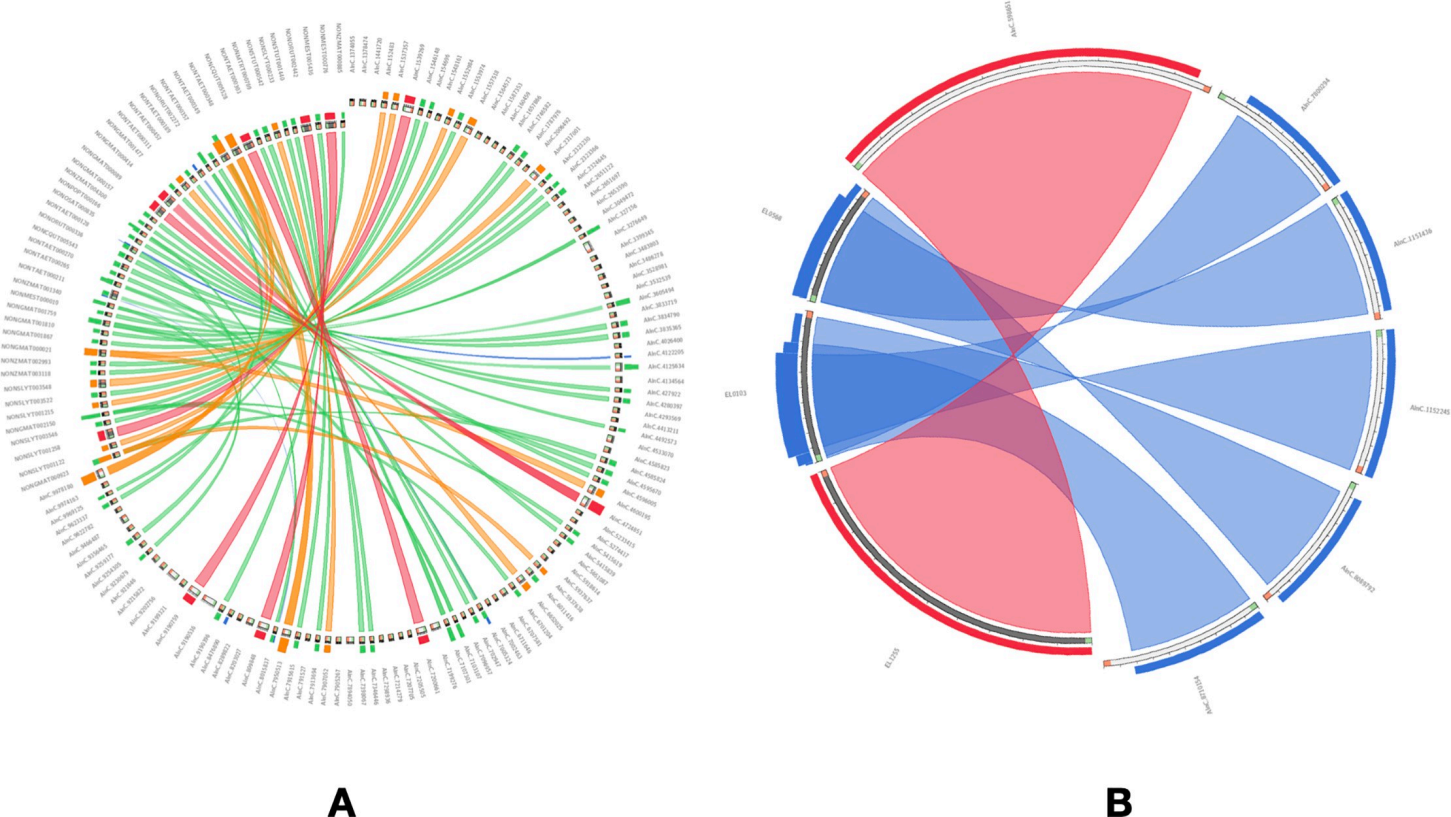
Circos plot showing significant BLAST hits of AlnC lncRNAs to, (A) NONCODE lncRNAs (only top 200 hits shown in the image), and (B) PLncDB (experimentally validated lncRNAs).

**Fig 3:**
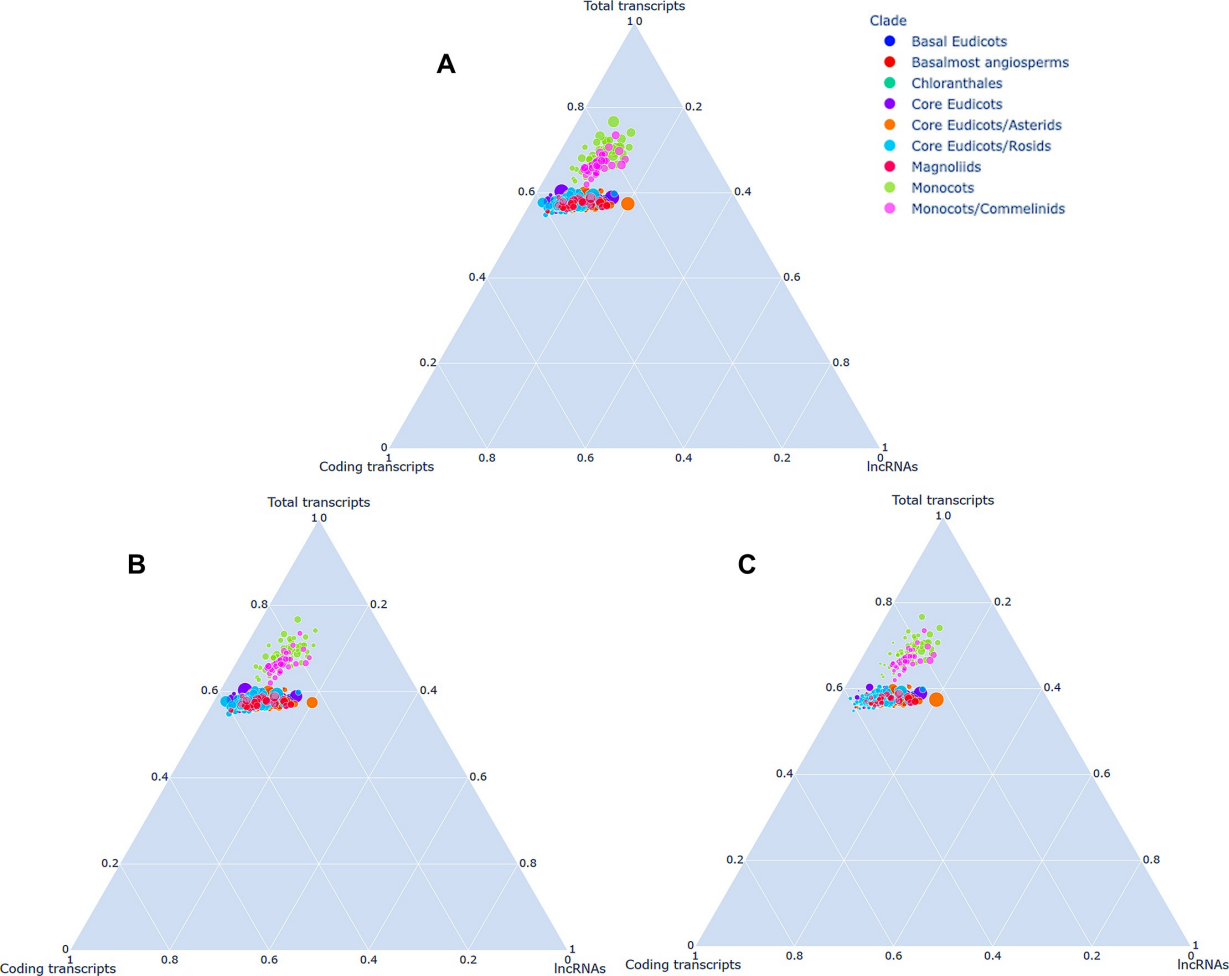
Correlation in total transcripts, coding transcripts and AlnC lncRNAs across clades. The bubble size represents the size of 1KP total transcripts, coding transcripts and AlnC annotated lncRNAs in (A), (B) and (C), respectively.

**Fig 4:**
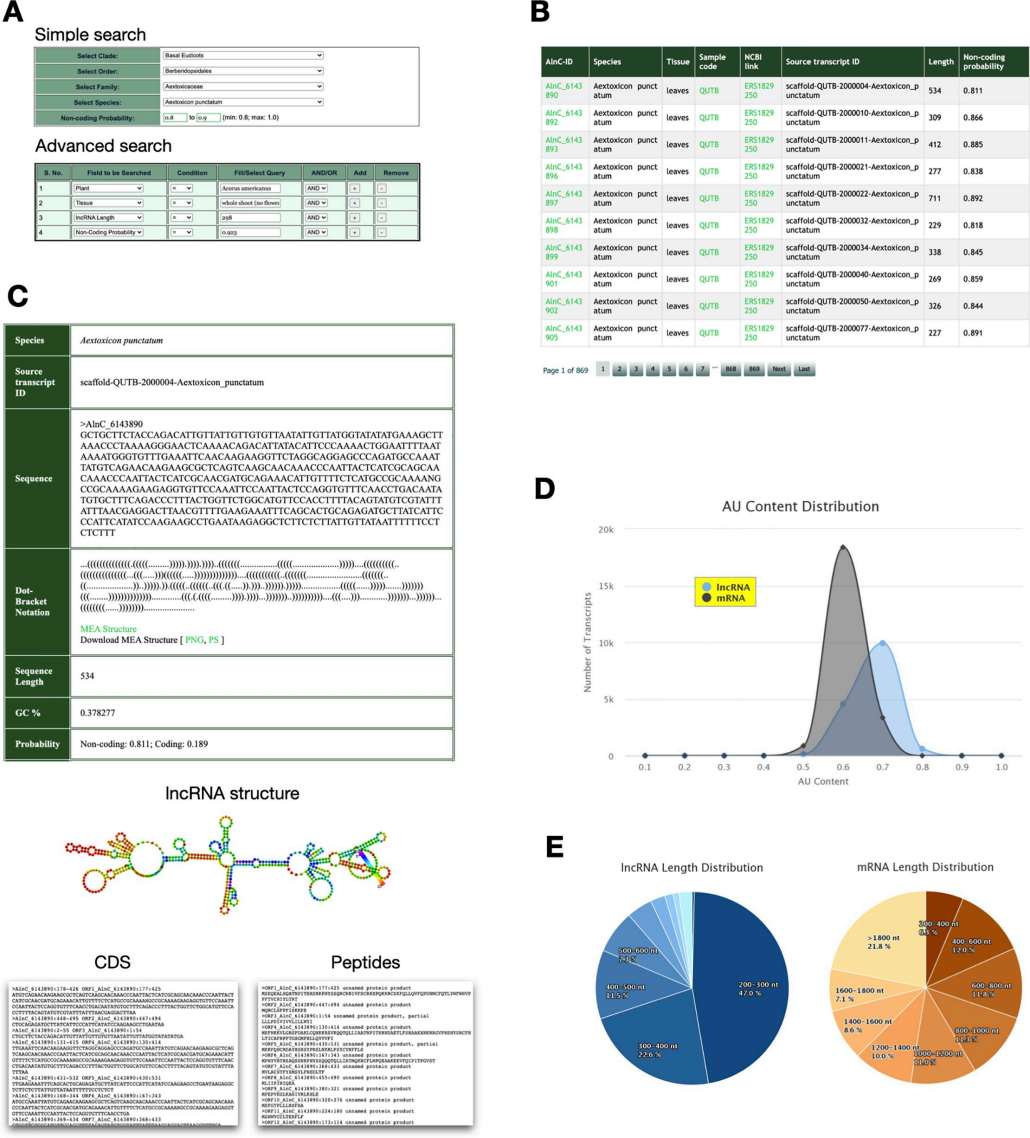
Screenshots of AlnC web interface. (A) Interface of simple search and advanced search modules. (B) Results page showing a table view of all lncRNA entries of the plant species *Aextoxicon punctatum*. (C) Sequence features including primary sequence information, secondary structure and possible sORFs as well peptides are displayed for lncRNA entry AlnC_6143890. (D) Percentage composition of AU content lncRNAs relative to mRNAs. (E) Pie chart representation of length-wise distribution of Aextoxicon *punctatum* lncRNAs and mRNAs.

We organised and compiled a collection of 1,08,55,598 lncRNAs from 809 samples available in the 1 KP project, which functions as a comprehensive lncRNA catalogue of 682 flowering plants stored in the AlnC platform (‘Statistics’ section of webpage). No other data repositories on lncRNAs on this scale exist, and most lncRNAs of the species included in AlnC belong to poorly studied taxa, rendering AlnC of wide interest among plant researchers (Fig 1(B) and 1(C)). In AlnC, the newly identified lncRNAs of flowering plants across clades ranged in size from 200 to 7633 nt with an average length of 405 nt (Fig 1(B)). We found the median length of identified lncRNAs is smaller than the median length of the coding sequences (Figs 1(B) and 4(E)). Moreover, most lncRNAs (66%) were less than 400 bp in length whereas only 2.9% lncRNAs were more than 1000 bp in length. We found the AU content of AlnC lncRNAs varied from 50–90% with an average of 75% in comparison to the coding transcripts which ranged 30–80% with an average of 60% (Fig 1(D)). Most lncRNAs contained more than 75% AU content, and the analysis implies the richness of AU than that in coding sequences [29–31]. Fig 1 and Table 1 provide a brief description of lncRNA entries in AlnC. Our attempts to identify ortholog relationships using BLASTn search (>70% identity, ± 50nt alignment length of subject/query lncRNA sequence, and e-value cutoff 0.01) resulted in significant hits, however, we could only identify a moderate number of AlnC annotated lncRNAs identical to those stored in two major databases, NONCODE (v6.0) and PLncDB (v2.0) (Fig 2) [13, 32]. This could be apparent differences in the number of lncRNAs in these database, but it also suggests certain conservation across species. The list of significant hits to NONCODE lncRNAs (384 hits), and experimentally validated lncRNAs available in Plncdb (6 hits) is tabulated in S1 Table. Further, we recognised the relationship between 1KP total transcripts, coding transcripts and AlnC annotated lncRNAs across clades using ternary graphs on the basis of size attributes to each axis separately (1 KP total transcripts, coding transcripts and lncRNAs in Fig 3A–3C, respectively). The ternary plot of AlnC annotated lncRNAs to 1 KP assembled transcripts and protein-coding transcripts enabled us to identify two distinct clusters of monocot and dicots, respectively (Fig 3). We observed proportional discovery rate of lncRNAs with respect to the 1KP assembled transcripts and also found out that the ratio of total transcripts to coding transcripts is smaller in dicot species whereas the reverse trend in case of monocots.

**Table 1:** Summary of annotated lncRNAs across higher-level clades in AlnC database.

Clade	Number of Species	Number of Samples	Number of lncRNAs
**Basal Eudicots**	33	55	835185
**Basalmost angiosperms**	8	8	281514
**Chloranthales**	2	2	46074
**Core Eudicots**	95	116	1828555
**Core Eudicots/Asterids**	217	242	3656812
**Core Eudicots/Rosids**	201	250	2627218
**Magnoliids**	26	27	383010
**Monocots**	61	64	829121
**Monocots/Commelinids**	39	45	568109
**Total**	682	809	10855598

### AlnC query and search platform

#### Search options

Current release of AlnC provides two query interfaces—(a) Simple Search, and (b) Advanced Search (Fig 4(A)). ‘Simple search’ allows users to perform quick searches for lncRNAs based on taxonomic rank (Clade, Order, Family, Species) and non-coding probability score (min: 0.8; max: 1.0) while ‘Advanced search’ provides enhanced query functionality using logical operators (AND/OR/> = /< =). Consequently, a list of potential lncRNAs will be displayed in the result page with the options to download and save search results. The search results display the entries of the chosen species covering the basic meta details of the 1KP sample code with links describing the sample information, including sample preparation, sample supplier, sample extractor and tissue type, NCBI ID showing experimental sample library and run data, source transcript ID from which lncRNA is annotated, lncRNA length and non-coding probability score (Fig 4(B)). The users can take advantage of AlnC ID link to view and navigate to a detailed record of a lncRNA providing primary lncRNA features, secondary structure and other accessory information.

#### lncRNA details webpage

Each ‘lncRNA details’ page of AlnC enables access to lncRNA and its structure information (Fig 4(C)). This detail webpage is divided into two parts: the first section contains basic details of lncRNA sequence (including species name of annotated lncRNA, source transcript ID, length, GC content, and coding/non-probability), and particulars of secondary structure in dot-bracket notation with a provision for the user to view and download the structure; second section displays ORFs and conceptual translation products of lncRNA sequence. Further, these peptides can be checked for possible functional activity with the BLAST option to the PlantPepDB database (contains 3,848 unique entries categorized into 9 major functional categories) [33]. All the associated annotations of a lncRNA entry can be downloaded in a tabular form.

#### BLAST module

BLAST feature was incorporated into AlnC web user interface to find regions of similarity between the user input and AlnC lncRNAs using BLASTn option. The default BLAST search enables searching for lncRNA transcript models of all angiosperm species in AlnC, and besides searches can also be limited by specifying species/clade/order/family and/or e-value cut-offs. The BLAST search output includes pairwise alignment, a report with BLAST hits based upon alignment scores and other measures of statistical significance.

#### LncRNA vs mRNA module

This module allows the interactive visualisation and comparison of the length-wise distribution as well as the AU percent of the annotated lncRNA transcripts relative to the protein-coding transcripts in multiple samples for each species (Fig 4(D) and 4(E)). This webpage also enables users with options to download these comparison plots in multiple image file formats.

#### Download AlnC data

Sequences can be searched and downloaded from the AlnC archive, and the full AlnC collection can also be found on the download page. The download webpage in AlnC provides access to both the hierarchical bulk download and the species-wise download in FASTA file format.

### Submit data

As there are sizeable researchers working on several flowering plants, we created a user form to submit any information or data with regard to angiosperm species lncRNAs. This will allow us to develop, upgrade and manage AlnC on a regular basis. The related lncRNA data, research results and publications can be submitted using the form provided on the ‘submit data’ section of webpage. If found to be relevant, the received data will be curated manually and appended to AlnC. All submitted lncRNA data will be processed in compliance with the AlnC standard pipeline mentioned in Fig 1(A) and manually curated. We encourage submissions to AlnC curators as this will drive our plans to include additional species in the future.

## Conclusion and future prospects

In this research work, we used transcriptome data from 682 flowering plants, most of which had no genomic information and/or no documented lncRNA studies prior to this work. This, in our view, is a key feature of AlnC as it was developed with the primary goal of facilitating lncRNA studies in various Angiosperm organisms. We used an analysis workflow tailored for plants, but it could also be used for RNA-seq-based lncRNA identification in non-plants species. The workflow provides a machine learning (ML)-based bioinformatics pipeline for identifying high-confidence lncRNAs across Angiosperm organisms, which differs from other annotation methods used in already available lncRNA databases (such as CATATAdb) [10]. This method produced a large number of putative lncRNA transcripts, which were then organised and catalogued in the AlnC web interface. AlnC covers information from 1,08,55,598 lncRNA loci derived from 809 samples and provides a user-friendly platform for browsing, searching and accessing all annotated lncRNAs via simple and interactive web pages. AlnC includes lncRNAs with evidence of non-coding RNA probability score and allows further exploration of sORFs alongside other primary lncRNA features, thus providing researchers with functional capability to leverage AlnC data and information on their individual projects through our web interface. With this research work, we attempted to develop a first-ever database covering the largest number of confident lncRNA entries of wide-range plant species, including those with no information on lncRNA of any sort. Although it is clear that several plants belonging to Angiosperms are still to be discovered and transcriptomes are waiting to be studied by individual research groups, AlnC will continue to be updated. At the same time, AlnC will strive to periodically search freely accessible databases, and other forms of documentation to gather useful information for annotated lncRNAs, and add additional functionality to enhance user engagement. It is our intention that AlnC will move forward to provide new databases representing additional species as well as to fine-tune and optimise the annotations currently available. We will also aim to focus on the new pipeline for the development of lncRNA annotations as the lncRNA biology research progresses. All in all, AlnC will strive to continue to be in line with the lncRNA community, and remain to serve useful lncRNA data in future.

## Supporting information

S1 Table List of significant hits of AlnC annotated lncRNAs to lncRNAs available at NONCODE and PlncDB databases. (XLSX)
